# Targeting Wnt Signaling for Gastrointestinal Cancer Therapy: Present and Evolving Views

**DOI:** 10.3390/cancers12123638

**Published:** 2020-12-04

**Authors:** Moon Jong Kim, Yuanjian Huang, Jae-Il Park

**Affiliations:** 1 Department of Experimental Radiation Oncology, The University of Texas MD Anderson Cancer Center, Houston, TX 77030, USA; mkim312@mdanderson.org (M.J.K.); yhuang14@mdanderson.org (Y.H.); 2 Graduate School of Biomedical Sciences, The University of Texas MD Anderson Cancer Center and Health Science Center, Houston, TX 77030, USA; 3 Program in Genetics and Epigenetics, The University of Texas MD Anderson Cancer Center, Houston, TX 77030, USA

**Keywords:** Wnt signaling, β-catenin, cancer, gastrointestinal cancers, therapeutic targeting of Wnt signaling, β-catenin paradox, molecular targeting

## Abstract

**Simple Summary:**

Therapeutic targeting of Wnt has long been suggested for gastrointestinal (GI) cancer treatment because deregulation of Wnt signaling is associated with GI cancers. However, therapeutic targeting of Wnt is still challenging because of the pleiotropic roles of Wnt signaling in the human body. Thus, targeting strategies of Wnt signaling are continuously evolving. The current flows of targeting Wnt signaling for cancer treatment are focused on increasing the specificity of drugs and combinatory treatment with other cancer drugs that minimize side effects and increase efficacy. Additionally, increased knowledge about the β-catenin paradox has expanded the cases that can be treated with Wnt targeting therapy, not strictly considering Wnt upstream and downstream mutations. Here, we discuss these evolving views of targeting Wnt signaling and describe examples of current clinical trials.

**Abstract:**

Wnt signaling governs tissue development, homeostasis, and regeneration. However, aberrant activation of Wnt promotes tumorigenesis. Despite the ongoing efforts to manipulate Wnt signaling, therapeutic targeting of Wnt signaling remains challenging. In this review, we provide an overview of current clinical trials to target Wnt signaling, with a major focus on gastrointestinal cancers. In addition, we discuss the caveats and alternative strategies for therapeutically targeting Wnt signaling for cancer treatment.

## 1. Introduction

Evolutionarily conserved Wnt signaling was initially identified in *Drosophila* (Wingless) and the mammalian system (Int-1) [1,2]. Wnt signaling has been extensively studied, revealing its pivotal roles in orchestrating embryonic development, tissue homeostasis, and regeneration [3,4,5]. Notably, the deregulation of Wnt signaling is associated with many human diseases, including cancers [6]. Therefore, the manipulation of Wnt signaling has gained attention as a means of disease treatment and prevention [7,8].

Although it has been confirmed in in vitro and in vivo cancer studies that targeting Wnt signaling has drastic tumor-suppressing effects, no targeted drugs have been successively advanced to clinical applications to date [7,8,9]. This is mainly because Wnt signaling plays essential roles in maintaining a broad range of physiological events [3,4,5]. Therefore, blocking Wnt signaling has detrimental impacts on tissue homeostasis and regeneration. In this review, we discuss current views on therapeutically targeting Wnt signaling and describe related clinical trials in gastrointestinal (GI) cancer.

## 2. Wnt Signaling

**Figure 1:**
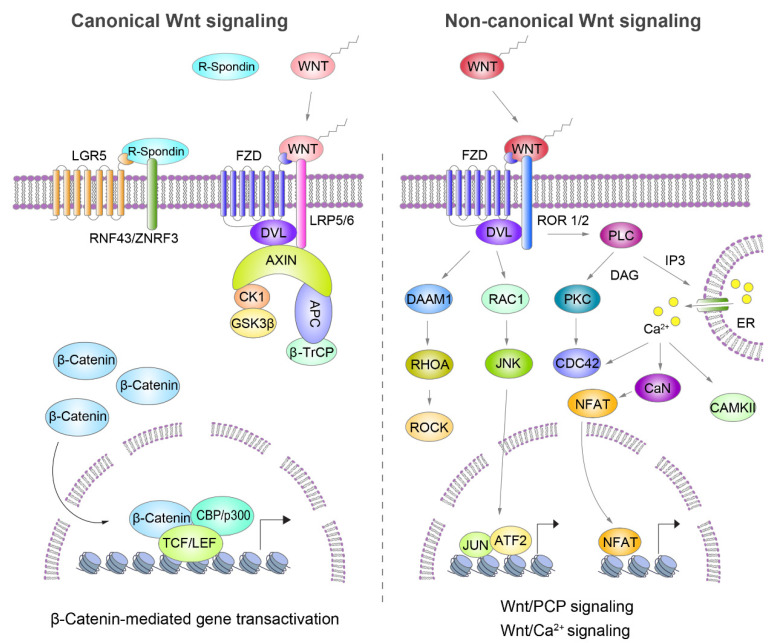
General view of canonical and non-canonical Wnt signaling. The switch of the canonical Wnt/β-catenin signaling pathway depends on the subcellular location of β-catenin. The stability of β-catenin is controlled by the destruction complex, consisting of AXIN, APC, CK1, and GSK3. In the absence of WNT ligands, cytoplasmic β-catenin is first phosphorylated by CK1 at Ser45 residue, followed by GSK3 phosphorylation at the Thr41, Ser37, and Ser33 residues. Next, the phosphorylated motif of β-catenin acts as a docking site for βTrCP, which induces the final ubiquitin-mediated degradation of β-catenin (Wnt off). When WNT ligands bind to Frizzled receptors (FZDs) and low density lipoprotein receptor-related protein co-receptor 5/6 (LRP 5/6), the destruction complex is recruited to the plasma membrane, triggering the translocation of β-catenin into the nucleus and activating its downstream target genes via binding directly to the TCF/LEF transcription factor family (Wnt on). Wnt/PCP signaling involves the triggering of a cascade that contains small GTPases RHOA (transforming protein RhoA) and Ras-related C3 botulinum toxin substrate 1 (RAC1), activating Rho-associated protein kinases (ROCKs) and JUN N-terminal kinases, respectively. Wnt/Ca^2+^ signaling involves the activation of phospholipase C, which in turn triggers the release of Ca^2+^ from intracellular stores and the activation of effectors such as calcium- or calmodulin-dependent protein kinase II, protein kinase C, and calcineurin (CaN). Next, CaN activates the nuclear factor of activated T cells, activating the transcription of downstream target genes.

Wnt signaling is an autocrine and paracrine signal-transducing module that is activated by lipid-modified WNT ligands and their receptors [10,11]. In humans, 19 WNT ligands and 18 receptors and coreceptors have been identified [10,12]. The Wnt ligand–receptor interaction activates a downstream cascade in a β-catenin-dependent or -independent manner [13] (Figure 1).

β-catenin is an Armadillo repeat protein that is mainly associated with E-cadherin at the inner plasma membrane. The β-catenin level is tightly regulated by the protein destruction complex, which is composed of the axis inhibitor (AXIN1), adenomatous polyposis coli (APC), casein kinase 1 (CK1), glycogen synthase kinase 3 (GSK3), and β-transducin repeat-containing protein (βTrCP) and induces β-catenin degradation through phosphorylation-mediated ubiquitination [11,14,15,16,17]. In β-catenin-dependent Wnt signaling (canonical Wnt signaling), the destruction complex is sequestered upon WNT ligand stimulation and disrupted by the formation of the WNT-receptor-disheveled (DVL) complex [18], resulting in the stabilization and nuclear translocation of β-catenin [19]. Next, nuclear β-catenin interacts with the TCF/LEF transcription factor family (TCF7, LEF1, TCF7L1, and TCF7L2), which recruits coactivators to transactivate downstream target genes [20,21,22,23]. β-catenin-independent Wnt signaling (also referred to as non-canonical Wnt signaling) activates downstream modules through the planar cell polarity (Wnt/PCP) pathway or Wnt/Ca^2+^ signaling pathway [10] (Figure 1).

In the Wnt/PCP pathway, the binding of WNT-FZDs triggers a cascade involving small GTPases RHOA (transforming protein RhoA) and RAC1 (Ras-related C3 botulinum toxin substrate 1), which in turn activates ROCKs (Rho-associated protein kinases) and JUN-N-terminal kinases, respectively [10,24,25]. It mainly regulates cell polarity, cell motility, and morphogenetic movements [10,24,25]. In the Wnt/Ca^2+^ signaling pathway, the binding of WNT-FZDs activates phospholipase C (PLC), which in turn triggers the release of Ca^2+^ from intracellular stores and the activation of effectors such as calcium- and calmodulin-dependent protein kinase II (CAMKII), protein kinase C (PKC), and calcineurin (CaN) [10,26]. CaN activates the nuclear factor of activated T cells, which regulates the transcription of the genes that control cell fate and cell migration [10,26]. Although both β-catenin-dependent and -independent Wnt signaling are involved in tumorigenesis, β-catenin-dependent Wnt signaling is relatively well defined in various cancer models. In line with this, current pharmacological trials targeting Wnt signaling have mainly focused on β-catenin-dependent Wnt signaling.

## 3. Wnt Signaling Alteration in GI Cancers

Hyperactivation of Wnt signaling is frequently observed in GI cancers, including colorectal cancer (CRC), hepatocellular carcinoma, gastric cancer, and pancreatic cancer. Approximately 90% of CRC demonstrates Wnt signaling-related gene alterations [27]. More than 70% of the genetic alterations in CRC are *APC* mutations [27,28]. Unlike CRC, *APC* mutations are rare in hepatocellular carcinoma. Hepatocellular carcinoma mainly displays *CTNNB1* mutations (20–35%) [29], *AXIN1* mutations (8–15%) [30], and Frizzled-7 (*FZD7*) overexpression (90%) [31]. In addition to mutations in the negative feedback regulator of the FZD receptor, the E3 ubiquitin-protein ligases *ZNRF3* and *RNF43* and their ligands, R-spondins (RSPOs), are frequently observed in pancreatic and gastric cancers [32,33].

## 4. Therapeutically Targeting Wnt Signaling in GI Cancer

Targeting Wnt signaling for cancer treatment normalizes the hyperactivated Wnt signaling that promotes cancer progression. For this purpose, many targeting strategies have been evaluated, including the inhibition of Wnt ligands and receptors or coreceptors, restoration of the destructive complex, and inhibition of β-catenin/β-catenin-dependent transcriptional machinery. Although these approaches have not been studied in phase III clinical trials or used clinically, dozens of Wnt-targeting agents are currently being evaluated in phase II clinical trials (Table 1). These important phase II clinical trials include LGK974, genistein, Foxy-5, DKN-01, niclosamide, PRI-724, and chloroquine/hydroxychloroquine.

**Figure 2:**
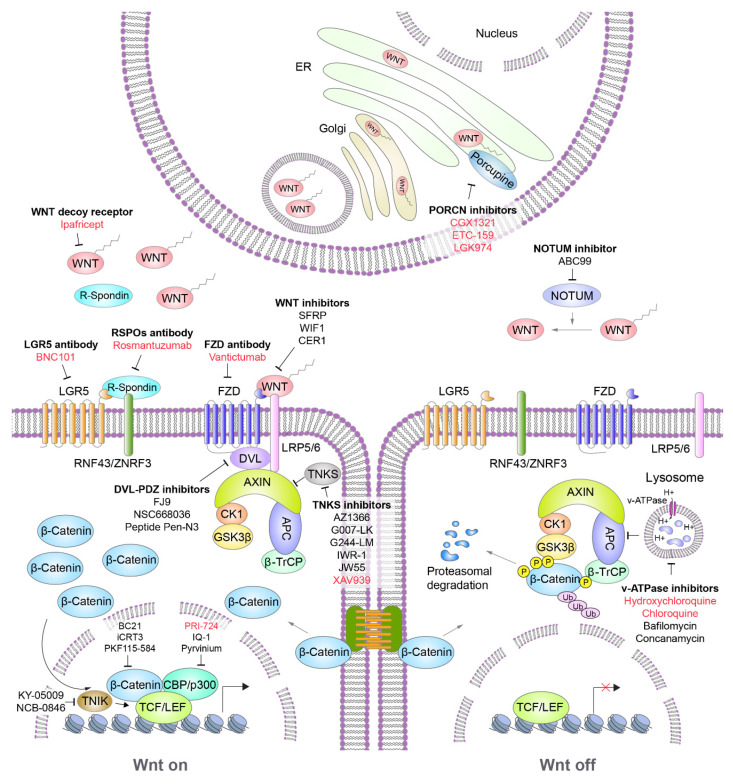
Wnt targeting agents for the Wnt/β-catenin signaling pathway. Wnt targeting agents for GI cancers mainly focus on the inhibition of the key molecules in Wnt/β-catenin signaling, such as inhibiting WNT ligands (ipafricept, LGK794), inhibiting Wnt receptors/coreceptors (vantictumab, rosmantuzumab), stabilizing the destruction complex (AZ1366, hydroxychloroquine), and inhibiting β-catenin-dependent transcriptional machinery (MSAB, PRI-724).

In the next section, we provide an overview of the known and potential agents that target Wnt signaling, especially for GI cancers; we also describe their mechanisms of action and related clinical trials (Table 2). All potential agents that inhibit Wnt signaling are listed in Table 3. In addition, the molecular targets of representative Wnt inhibitors on WNT signaling are illustrated in Figure 2.

## 5. Targeting WNT Ligands

### 5.1. Inhibiting WNT Ligands

Ipafricept (OMP-54F28) is a recombinant receptor that is comprised of the cysteine-rich domain of FZD8 fused to the human IgG1 Fc domain; it inhibits Wnt signaling by neutralizing WNT ligands [54]. Three trials evaluated ipafricept and its combination therapies (Table 2). A phase I trial evaluated the best dosage of ipafricept and revealed grade 1–2 adverse events (AEs), including dysgeusia, decreased appetite, fatigue, and muscle spasms [36]. Another phase I trial evaluated ipafricept combined with nab-paclitaxel and gemcitabine in metastatic pancreatic cancer and revealed grade ≥ 3 AEs, including increased aspartate aminotransferase, nausea, maculopapular rash, vomiting, and decreased white blood cells [37].

Secreted frizzled-related proteins (SFRPs) bound directly to WNTs via the cysteine-rich domain, preventing the WNT–FZD interaction [55,56,57]. SFRPs also form dimers with FZDs via the respective cysteine-rich domain to activate or inhibit WNT3A/β-catenin signaling, depending on their concentration [58]. In the nucleus, SFRPs act as biphasic modulators of β-catenin-mediated transcription, which promotes TCF7L2 recruitment and transactivation of cancer stem cell-related genes by binding to the β-catenin’s C-terminus; however, they suppress transcriptional activities by binding to the N-terminus [59]. The phase II trial evaluated genistein, an *SFRP2* silencer inhibitor, in combination with FOLFOX and bevacizumab in metastatic CRC; the study revealed mild AEs, including headaches, nausea, and hot flashes (Table 2) [35]. In addition, Wnt inhibitory factor 1 directly binds to WNTs through the Wnt inhibitory factor domain and prevents WNTs from transducing Wnt signaling [60]. Cerberus also binds to and inhibits WNT8, inhibiting Wnt signaling [61]. However, no agents mimicking Wnt inhibitory factor 1 and Cerberus have been identified.

### 5.2. Targeting Lipid Modification of WNT Ligands

The palmitoylation of WNT ligands by the protein-serine O-palmitoleoyltransferase porcupine in the endoplasmic reticulum [62] is essential for the maturation and extracellular secretion of WNT ligands. The palmitoylated WNT ligands bind to Wntless homolog in the Golgi and are ferried to the plasma membrane via secretory exosomes [63]. Porcupine inhibitors (CGX1321, ETC-159, and LGK974 [WNT794]), which suppress Wnt signaling by blocking the secretion of WNT ligands, are currently being evaluated in clinical trials (Table 2). A phase I trial evaluated the best dosage of ETC-159 and revealed well-tolerated AEs, including vomiting, anorexia and fatigue, dysgeusia, and constipation [34]. The lipid modification of WNTs can be enzymatically removed by the palmitoleoyl-protein carboxylesterase NOTUM, thereby inhibiting Wnt signaling [64]. The NOTUM inhibitor, ABC99, is effective in the treatment of benefiting osteopenia and osteoporosis by enhancing Wnt signaling (Table 3) [64,65]. However, no agents have been identified that mimic NOTUM to inhibit GI cancers. Alternatively, metalloprotease TIKI1 (Trabd2a) acts as a protease to cleave eight amino acid residues of WNTs, resulting in oxidized WNT oligomers with minimized receptor binding capability in frogs [66,67]. However, no agents have been identified that mimic the impact of TRABD on Wnt signaling in humans.

## 6. Targeting Wnt Receptors and Co-Receptors

### 6.1. Antibodies against FZDs

Vantictumab (OMP-18R5) is a monoclonal antibody that binds to FZD 1, 2, 5, 7, and 8 and inhibits Wnt signal transduction [54]. A phase I trial evaluating the best dosage of vantictumab combined with nab-paclitaxel and gemcitabine in metastatic pancreatic cancer was terminated because of the increased risk of bone fracture [39]. Moreover, FZD5 has been identified as a dominant FZD receptor in RNF43-mutant pancreatic cancer cells and may be a therapeutic index [68]. However, no agents targeting FZD5 have been introduced.

### 6.2. Mimetic Agents Binding to FZDs

Initially, WNT5A was classified as a non-canonical Wnt family member. It activates Wnt/Ca^2+^ signaling by stimulating intracellular Ca^2+^ flux in zebrafish and frogs [69,70,71,72]. In 2006, Mikels et al. found that WNT5A also activates canonical Wnt signaling via FZD4 and LRP5 [73]. Intriguingly, WNT5A additionally inhibits WNT3A-induced canonical Wnt signaling via FZD2 and tyrosine-protein kinase transmembrane receptor ROR2 [73,74]. Therefore, the function of WNT5A is considered not limited to the field of Wnt signaling and is more dependent on the context of receptors. Foxy-5, a WNT5A peptide mimic, reduces the metastatic capacity of invasive breast cancer via epithelial discoidin domain-containing receptor 1 (DDR1), which decreases the motility and the invasive potential of breast epithelial cells [75,76,77]. However, whether these mechanisms are also true in GI cancers remains unknown. Foxy-5 is being evaluated in phase I-II clinical trials of metastatic CRC, but no results have been published [38] (Table 2).

### 6.3. Inhibiting LRP5/6

Given that dickkopf-related protein 1 (DKK1) inhibits Wnt signaling through its direct binding to LRP5/6 [78,79], DKK1 was initially considered a tumor suppressor in the β-catenin-dependent context. Conversely, several studies have shown that DKK1 promotes tumor cell proliferation, metastasis, and angiogenesis, which might be mediated by β-catenin-independent signaling [80,81,82,83,84,85,86]. One available explanation is that DKK1 interacts with both glypican4 (GPC4) and the LRP/KREMEN complex to induce the endocytosis of LRP5/6, transforming the biochemical properties of FZDs and their cytoplasmic components from the Wnt/β-catenin pathway to the Wnt/PCP signaling axis [87,88]. This mechanism activating β-catenin-independent signaling and inhibiting β-catenin-dependent signaling was validated in zebrafish and frogs [87,88].

On the basis of the tumorigenic role of DKK1, DKN-01, a DKK1 monoclonal antibody, was developed for cancer therapy. Four trials evaluating DKN-01 and its combination therapies are ongoing (Table 2). A phase I trial assessing DKN-01 combined with paclitaxel in advanced esophageal and gastroesophageal junction cancer revealed that 35% of patients experienced a partial response [40,89]. Another phase I trial of the best dosage of DKN-01 combined with gemcitabine and cisplatin in advanced biliary cancer revealed that 33.3% of patients experienced a partial response [41]. Sclerostin domain-containing protein 1 can activate or inhibit Wnt signaling by mimicking WNT ligands or by competing with WNT8 for binding to LRP6, respectively [90,91]. However, no agents simulating sclerostin domain-containing protein 1 have been identified.

### 6.4. Accelerating the Degradation of FZD/LRP Receptors

Secreted RSPOs (RSPO1-3) and their receptors, RNF43/ZNRF3, are required to potentiate Wnt signaling in various development and tissue homeostasis contexts [92,93,94]. In addition, leucine-rich repeat-containing G-protein-coupled receptors (LGRs, LGR4-6) are required for the interaction between RSPOs and their receptors [92]. Without RSPOs and LGRs, RNF43/ZNRF3 induces the internalization and degradation of FZD receptors and negatively regulates Wnt signaling [92,95,96].

A phase I trial evaluated the best dosage of rosmantuzumab (OMP-131R10), a monoclonal antibody against RSPO3, for metastatic CRC; no results have been published (Table 2). BNC101, a monoclonal antibody against LGR5, demonstrated antitumor activity in multiple CRC patient-derived xenografts, but the clinical trial was terminated (Table 2) [97]. Niclosamide, a teniacide in the anthelmintic family, promotes FZD1 endocytosis, inhibiting WNT3A/β-catenin signaling in CRC and osteosarcoma and inducing LRP6 degradation in prostate and breast cancer [98,99,100]. The NIKOLO trial and NCT02687009 have been evaluating niclosamide in CRC (Table 2). The NIKOLO trial has revealed no drug-related AEs [43].

## 7. Targeting the Destruction Complex

### 7.1. Inhibiting the DVL–FZD Interaction

In the presence of WNT ligands, DVLs bind to the cytoplasmic domain of FZDs via the PDZ (PSD95, DLG1, and ZO1) domain, which provides a platform for the interaction between the LRP’s tail and AXIN to recruit the destruction complex onto the cytoplasmic membrane [101,102]. This process inhibits destruction complex-mediated β-catenin protein degradation [93]. Several inhibitors (compound 3289-8625, FJ9, NSC668036, and peptide Pen-N3) that directly inhibit DVL binding with FZDs are currently being evaluated in preclinical studies (Table 3) [103,104,105,106].

### 7.2. Stabilizing AXIN

Tankyrase is a member of the poly ADP-ribose polymerase superfamily of proteins which mediates the PARsylation and proteasomal degradation of AXIN [107,108]. Tankyrase inhibitors (AZ1366, G007-LK, G244-LM, IWR-1, JW55, and XAV939) that stabilize AXIN and activate the destruction complex are being evaluated in preclinical studies (Table 3) [109,110,111,112,113]. The E3 ubiquitin-protein ligase SIAH, a potent activator of Wnt signaling, promotes the ubiquitination and proteasomal degradation of AXIN by interacting with a VxP motif in the GSK3-binding domain of AXIN [114]. Ubiquitin carboxyl-terminal hydrolase 7 (USP7), a potent negative regulator of Wnt/β-catenin signaling, promotes the deubiquitination and stabilization of AXIN by interacting with AXIN through its TRAF domain [115]. However, no agents that inhibit SIAH or mimic USP7 have been identified.

### 7.3. Stabilizing APC

Transmembrane protein 9 (TMEM9) binds to and facilitates the assembly of vacuolar-type H^+^-ATPase (v-ATPase), resulting in enhanced vesicular acidification and trafficking for subsequent lysosomal degradation of APC and hyperactivation of Wnt/β-catenin signaling [116]. Conversely, pharmacological targeting of v-ATPase using bafilomycin, concanamycin, hydroxychloroquine, or KM91104 inhibits Wnt/β-catenin signaling and suppresses intestinal tumorigenesis (Table 3) [116]. Twenty trials are currently evaluating v-ATPase inhibitors (Table 2). A phase II trial assessing hydroxychloroquine combined with gemcitabine in unresectable pancreatic cancer revealed no dose-limiting AEs [46]. Another phase II trial revealed an increased overall response rate (38.2 vs. 21.1%; *P* = 0.047) but no survival benefits (hazard ratio, 1.14; 95% CI, 0.76–1.69; *P* = 0.53) when adding hydroxychloroquine to combination therapy with nab-paclitaxel and gemcitabine for advanced pancreatic cancer [50].

### 7.4. Activating CK1 and GSK3

CK1 and GSK3 sequentially phosphorylate β-catenin to induce the ubiquitination and proteasomal degradation of β-catenin [16]. Therefore, CK1 and GSK3 activators likely reduce the level of β-catenin that translocates into the nucleus, consequently inactivating Wnt signaling. pyrvinium, a CK1 activator that binds to the C-terminal regulatory domain of its isoform CK1A1, has been introduced, but it has not been evaluated in clinical trials (Table 3) [117]. In addition, no GSK3 activators have been introduced.

## 8. Targeting β-Catenin and β-Catenin-Dependent Transcriptional Machinery

### 8.1. Promoting β-Catenin Degradation

Methyl 3-[[(4-methylphenyl)sulfonyl]amino] benzoate (MSAB) [12] binds to the Armadillo repeat domain of β-catenin and promotes its degradation [118]. NRX-252114, a protein–protein interaction enhancer, enhances the interaction between β-catenin and its cognate E3 ligase, potentiating the ubiquitination-mediated degradation of β-catenin [119]. No clinical trials have evaluated MSAB and NRX-252114.

### 8.2. Inhibiting the β-Catenin–TCF/LEF Complex

With its increased fold change, nuclear β-catenin replaces the transducin-like enhancer protein corepressor with coactivators by forming the β-catenin–TCF/LEF complex [93,120]. This complex transactivates Wnt target genes through its sequence-specific DNA binding and context-dependent interaction [121]. β-catenin-TCF/LEF complex inhibitors (BC21, iCRT3, and PKF115-584) were introduced in preclinical studies (Table 3) [122,123,124].

### 8.3. Manipulating TCF/LEF Phosphatases

TRAF2 and NCK-interacting protein kinase (TNIK) phosphorylates the serine 169 residue of TCF7L1 and the serine 154 residue of TCF7L2, acting as an activating kinase of the β-catenin-TCF/LEF transcriptional complex [125,126,127]. TNIK inhibitors (KY-05009 and NCB-0846) are being evaluated in preclinical studies [126,128] (Table 3). Serine/threonine-protein kinase NLK phosphorylates the threonine 155 and serine 166 residues of LEF1 and the threonine 178, 189 residues of TCF7L2, triggering their dissociation from DNA and inhibiting Wnt target gene transactivation [129,130]. Homeodomain-interacting protein kinase 2 (HIPK2) phosphorylates LEF1, TCF7L1, and TCF7L2 to dissociate them from DNA, which positively or negatively modulates Wnt/β-catenin signaling [131,132]. However, no agents targeting NLK and HIPK2 have been identified.

### 8.4. Inhibiting Coactivators

CREB-binding protein (CREBBP), histone acetyltransferase EP300, pygopus homolog (PYGO), and B-cell CLL/lymphoma 9 protein (BCL9) are coactivators that interact with the β-catenin–TCF/LEF complex [10]. PRI-724 competes with β-catenin to bind with CREBBP, suppressing the transcriptional activation of β-catenin target genes [133]. Three trials have been evaluating PRI-724, two of which were terminated or withdrawn because of low enrollment or a drug supply issue (Table 2). A phase I trial evaluating the best dosage of PRI-724 revealed grade 2 AEs, including diarrhea, bilirubin elevation, hypophosphatemia, nausea, fatigue, anorexia, thrombocytopenia, and alkaline phosphatase elevation [52]. Another phase I trial evaluating the best dosage of PRI-724 combined with gemcitabine as second-line therapy for advanced pancreatic cancer revealed grade ≥ 3 AEs, including abdominal pain, neutropenia, anemia, fatigue, and alkaline phosphatase elevation [53]. The inhibitors of EP300, PYGO, and BCL9 (IQ-1, pyrvinium, and carnosic acid, respectively) have been evaluated in preclinical studies (Table 3) [117,134,135]. In addition, SM08502, a CDC-like kinase (CLK) inhibitor that blocks the phosphorylation of serine/arginine-rich splicing factors and consequently disrupts spliceosome activity, has been shown to inhibit Wnt signaling in preclinical models [136,137,138]. A phase I trial evaluating SM08502 for advanced GI cancers is ongoing (Table 2).

## 9. Caveats in Targeting Wnt Signaling

### 9.1. Targeting Core Components of Wnt Signaling

The major caveat in Wnt targeting strategies is their detrimental side effects on normal cells in which Wnt signaling plays pivotal roles in tissue homeostasis and regeneration [3,4,5]. For example, intestinal stem cells replenish the intestinal epithelium every 3 to 4 days; this is tightly regulated by constitutively active Wnt signaling in the crypt bottom [139,140]. Inhibiting Wnt signaling disrupts intestinal homeostasis and induces the severe loss of the crypt-villi structure. Similarly, upon Wnt blockade, tissue homeostasis disruption also takes place in hair follicles, the stomach, and the hematopoietic system, where Wnt signaling is indispensable for the maintenance of stem cells and their niches [141,142,143]. Indeed, the treatment of the FZD inhibitor (vanctumab) and antagonist (ipafricept) leads to side effects, including tiredness, diarrhea, vomiting, constipation, bone metabolism disorders, and abdominal pain [36,54]. Wnt signaling is also required for tissue homeostasis and regeneration in the lungs, liver, skin, and pancreas [3,4,5]. Therefore, Wnt signaling targeting strategies need to be meticulously designed and evaluated on the basis of their specificity and efficacy, which is discussed in the next section.

### 9.2. Targeting Upstream vs. Downstream

Targeting the downstream effectors of Wnt signaling, e.g., β-catenin and TCF/LEF, might maximize Wnt signaling inhibition on the basis of signaling convergence into downstream gene regulation. However, targeting downstream Wnt signaling might also generate severe side effects by disrupting Wnt signaling in normal tissues. Conversely, targeting the upstream molecules of Wnt signaling, e.g., ligands and receptors, was initially considered ineffectual in cancer cells carrying mutations in Wnt signaling downstream (i.e., *APC* and β-catenin/*CTNNB1*) [93]. Intriguingly, accumulating evidence suggests that targeting Wnt signaling upstream is also effective independent of Wnt signaling downstream mutations. This evolving concept, the “β-catenin paradox”, is discussed below.

## 10. Evolving Views in Targeting Wnt Signaling

### 10.1. Cancer- and Tissue-Specific Wnt Signaling Targeting

Targeting cancer type- or tissue-specific Wnt signaling components or modulators may overcome the side effects of Wnt signaling blockade on normal tissues. For instance, specifically targeting the constitutively active form of β-catenin mutants may be ideal. A recent study found that small-molecule enhancers of mutant β-catenin and its E3 ligase (β-TrCP) interaction potentiate the ubiquitination-mediated degradation of mutant β-catenin [119], suggesting one possible approach to targeting the mutant form of β-catenin.

There are also several promising preclinical and clinical studies evaluating antibodies against RSPOs and LGRs, Wnt signaling amplifiers [42]. Since RSPOs and LGRs are differently expressed in different tissues and cancers [144,145], targeting them might diminish normal tissue damage. LGR5 has been suggested as a cancer stem cell marker [146,147], and targeting LGR5+ cells with anti-LGR5 antibody–drug conjugates suppressed tumor growth and metastasis in a preclinical model [145,148]. Anti-LGR5 therapy and anti-RSPO3 (rosmantuzumab) are currently being evaluated in phase I trials for the treatment of metastatic CRC (NCT02726334 and NCT02005315) (Table 2). RSPO3-LGR4-maintained Wnt signaling is essential for the stemness of acute myeloid leukemia, and the clinical-grade anti-RSPO3 antibody eradicated leukemia stem cells [149], which might be effective in GI cancer. The results of these studies indicate that blockage of cancer- or tissue-specific Wnt signaling components or regulators are viable options for GI cancer treatment.

### 10.2. Efficacy and Combination Therapy

An alternative method of overcoming limitations in Wnt signaling targeting strategies is to identify a safe dose that is highly effective but does not disrupt normal physiologic processes. A specific dose of LGK794 had lower severity of side effects with effective pharmacologic outcomes in a phase I clinical trial [7]. It is also noteworthy that different tissues showed different levels of Wnt signaling threshold in vivo [150], supporting the theory that localizing treatment is an alternative strategy to avoid toxicity and side effects.

In general, combination therapy is considered to result in more AEs. However, it does not always induce more AEs than does monotherapy. The incidences and degrees of AEs depend on various factors, such as the doses of single drugs, the timing of administration, the period of treatment, the supportive treatment, and the heterogeneity of the patients themselves. Thus, certain drug dose combinations may be more effective, with fewer AEs. Furthermore, monotherapy targeting one pathway does not guarantee complete anticancer activity because of multiple crosstalks and compensations by other signaling pathways. Although its efficacy may be counterbalanced by correspondingly increased toxicity, combination therapy that simultaneously targets several pathways might be more efficient. In addition, combination therapy is the most common approach to achieving survival benefits in clinical practice, and most promising phase III Wnt targeting trials use combination therapy.

ICG-001 and PRI-724 inhibit Wnt target gene expression by antagonizing CBP, a β-catenin coactivator [133,151]. PRI-724 was effective in a phase I clinical trial of PDAC when used in combination with gemcitabine (NCT01764477). Other cases include the combination of anti-FZD antibody with chemotherapy. Vantictumab (OMP-18R5) resulted in promising outcomes in the preclinical setting [152,153] and is currently being evaluated in phase I clinical trials for multiple cancers in combination with paclitaxel [154]. Ipafricept (OMP-54F28/FZD8-Fc) is being evaluated in a phase I clinical trial to treat advanced pancreatic cancer in combination with nab-paclitaxel and gemcitabine [36]. Although antibodies against pan-Wnts or pan-FZD were not tissue-specific, their combination in advanced solid tumors had promising effects [36,154]. In addition, as a neoadjuvant therapy, Foxy-5 is currently being evaluated in phase II trials for colon cancer, as described above (NCT03883802).

### 10.3. β-Catenin Paradox

The β-catenin paradox was introduced on the basis of heterogeneous Wnt signaling activity in CRC cells, carrying homogenous genetic alterations in *APC* or β-catenin/*CTNNB1* [155]. This observation was followed by discoveries of several Wnt signaling regulators and multiple crosstalks of Wnt/β-catenin signaling with MAPK and PI3K pathways [156,157,158,159,160,161,162,163,164,165]. Additionally, accumulating evidence suggests that the blockade of Wnt signaling upstream molecules suppresses tumor growth despite the presence of oncogenic mutations in Wnt signaling components [96,108,116,152,166,167], demonstrating the existence of additional regulatory modules in Wnt signaling, independent of genetic alterations. Additionally, truncated mutant APC remains partially functional to induce β-catenin protein degradation [116,167]. Moreover, the blockade of WNTs/RSPOs inhibits the growth of tumor cells that harbor *APC* mutations [96,116]. In line with this, Tankyrase inhibitor-stabilized AXIN protein suppresses the proliferation of CRC cells that carry constitutively active mutations in β-catenin or *APC* [108,110]. A recent gastric cancer mouse model study also revealed that vantictumab, the pan-FZD inhibitor, inhibits gastric adenoma growth independently of *APC* mutations [152]. Therefore, molecular targeting of the upstream molecules of APC and β-catenin might be promising in Wnt/β-catenin signaling-associated cancer.

### 10.4. Generalization of Wnt Targeting Therapy

Aberrant Wnt signaling is crucial for the potential clonal source of tumor cells and is considered an environmental and metastatic niche for tumor progression. Indeed, LGR5+ colon cancer cells are required for the formation of metastatic colonization in the liver [146]. A study using patient-derived pancreatic organoids revealed differing Wnt-niche dependency among organoids [168]. Furthermore, in a recent study of lung cancers that barely harbor Wnt mutations, Wnt signaling was shown to be required for lung cancer progression as a niche factor in a mouse lung adenocarcinoma model [169]. In that context, Wnt targeting by porcupine inhibitor, WNT794 (LGK794), revealed the suppression of lung tumor progression [169]. These results suggest that Wnt targeting therapy can be generalized to various types of non-Wnt-mutated cancers in which Wnt signaling has tumor-promoting or metastatic roles.

## 11. New Candidates for Targeting Wnt Signaling in GI Cancers

Several cancer-specific Wnt signaling regulators were identified in GI cancers. Amplification of USP21 deubiquitinase promotes pancreatic cancer cell growth and stemness via Wnt/β-catenin signaling [170]. RNF6, a CRC-upregulated E3 ligase, promotes CRC cell growth through the degradation of Tele3, a transcriptional repressor of the β-catenin/TCF4 complex [171]. Another deubiquitinase USP7 serves as a tumor-specific Wnt activator in *APC*-mutated CRC by promoting β-catenin deubiquitination [172]. Transcriptional coactivators of β-catenin, BCL9 and BCL9l, redundantly demonstrated CRC-specific upregulation, and their loss suppressed intestinal tumorigenesis in a mouse model [173]. BCL9 and BCL9l inhibitors were recently developed [135,174,175]. Targeting BCL9 and BCL9l has been suggested as a therapeutic approach to CRC-specific treatment. FZD5 mainly expressed in RNF43 mutated tumor cells was proposed as a molecular target for pancreatic cancer treatment [68]. Given that gut-specific knockout of *FZD5* is feasible in the mouse models [176,177], it is likely that targeting of FZD5 can be used in RNF43 mutated intestinal or gastric tumors. In addition, CRC-upregulated PAF/KIAA0101 hyperactivates Wnt/β-catenin signaling and accelerates tumorigenesis in vitro and in vivo [178,179]. As an amplifier of Wnt signaling, TMEM9 hyperactivates β-catenin via APC degradation to promote intestinal and hepatic tumorigenesis [116,166]. Of note, germline deletion of *Tmem9* or *Paf* did not display any discernible phenotypes, suggests that blockade of cancer-related Wnt signaling activators or amplifiers minimizes side effects in Wnt signaling targeting approaches.

Additionally, recent technological advances in organoids made it feasible to perform high-throughput chemical screening (clinical drugs or drug library) and genetic screening (gene knock-out or knock-down) of tumor organoids [180,181,182]. Moreover, patient-derived organoids become valuable resources to identify most effective drug(s) for precision medicine including pharmacogenomics [183,184,185]. Therefore, with the emergence of such new technology, it is anticipated that novel tumor-specific and druggable vulnerabilities related to Wnt signaling hyperactivation will be identified.

## 12. Conclusions

To date, many studies have reported the marked impact of molecular targeting of Wnt signaling on tumor suppression in preclinical settings. Despite the ongoing clinical trials, it is still imperative to overcome recurring pitfalls—catastrophic adverse effects on tissue homeostasis and regeneration. Like the sword of Damocles, targeting Wnt signaling poses a high risk but has significant potential in cancer therapy. With evolving concepts in Wnt signaling deregulation and manipulation, new and improved approaches, including molecular targeting of upstream signaling modules or cancer-specific regulators and combination therapy, are expected to open a new window of opportunity in the treatment of Wnt signaling-associated cancer.

## Figures and Tables

**Table 1:** Agents inhibiting Wnt signaling for GI cancers in phase II clinical trials.

Agent	Mechanism	Trial	Cancer
LGK974	PORCN inhibitor	NCT02278133	BRAF V600-mutated metastatic colorectal cancer
Genistein	*SFRP2* silencer inhibitor	NCT01985763	Metastatic colorectal cancer
Foxy-5	WNT5A mimic	Vermorken 2019	WNT5A-negative colon cancer
DKN-01	Monoclonal antibody against DKK1	NCT03645980; NCT04166721	Advanced hepatocellular carcinoma; Advanced gastroesophageal adenocarcinoma
Niclosamide	FZD1 inhibitor, LRP6 inhibitor	NCT02519582	Progressed colorectal cancer
PRI-724	β-catenin/CREBBP inhibitor	NCT02413853	Metastatic colorectal adenocarcinoma
Chloroquine	v-ATPase inhibitor	NCT02496741	Advanced solid malignancies, including intrahepatic cholangiocarcinoma
Hydroxy-chloroquine	v-ATPase inhibitor	NCT01006369, etc. (total 13 trials)	Advanced colorectal carcinoma; Advanced hepatocellular carcinoma; Advanced cholangiocarcinoma; Pancreatic adenocarcinoma

**Table 2:** Agents inhibiting Wnt signaling for GI cancers in clinical trials.

Trial	Agent	Mechanism	Design	Cancer	Interventions	Status
NCT02675946	CGX1321	Porcupine inhibitor	Phase I; Single group	Advanced GI cancers	CGX1321; CGX1321 + pembrolizumab	Recruiting
NCT03507998	CGX1321	Porcupine inhibitor	Phase I; Single group	Advanced GI cancers	CGX1321	Recruiting
Ng 2017 (NCT02521844) [34]	ETC-159	Porcupine inhibitor	Phase I; Single group	Advanced solid malignancies, including colorectal cancer, etc.	ETC-159; ETC-159 + pembrolizumab	Ongoing
NCT01351103	LGK974	Porcupine inhibitor	Phase I; Single group	Solid malignancies, including esophageal squamous-cell carcinoma, pancreatic adenocarcinoma, BRAF-mutated colorectal cancer, etc.	LGK974; LGK974 + spartalizumab	Recruiting
NCT02278133	LGK974	Porcupine inhibitor	Phase II; Single group	BRAF V600-mutated metastatic colorectal cancer with RNF43 mutations and/or R-spondin fusions	LGK974 + LGX818 + cetuximab	Completed
Pintova 2019 (NCT01985763) [35]	Genistein	*SFRP2* silencer inhibitor	Phase II; Single group	Metastatic colorectal cancer	Genistein + FOLFOX; Genistein + FOLFOX + bevacizumab	Completed
Jimeno 2017 (NCT01608867) [36]	Ipafricept (OMP-54F28)	WNT decoy receptor	Phase I; Single group	Solid malignancies, including pancreatic cancer, colorectal cancer, etc.	Ipafricept	Completed
Dotan 2019 (NCT02050178) [37]	Ipafricept (OMP-54F28)	WNT decoy receptor	Phase I; Single group	Metastatic pancreatic ductal adenocarcinoma	Ipafricept + nab-paclitaxel + gemcitabine	Completed
NCT02069145	Ipafricept (OMP-54F28)	WNT decoy receptor	Phase I; Single group	Advanced hepatocellular carcinoma	Ipafricept + sorafenib	Completed
NCT02020291	Foxy-5	WNT5A mimic	Phase I; Single group	Metastatic breast, colon, prostate cancer	Foxy-5	Completed
NCT02655952	Foxy-5	WNT5A mimic	Phase I; Single group	Metastatic breast, colon, prostate cancer	Foxy-5	Completed
Vermorken 2019 [38]	Foxy-5	WNT5A mimic	Phase II; Randomized; Parallel	WNT5A-negative colon cancer	Foxy-5 vs placebo	Recruiting
Davis 2019 (NCT02005315) [39]	Vantictumab (OMP-18R5)	Monoclonal antibody against FZDs	Phase I; Single group	Metastatic pancreatic ductal adenocarcinoma	Vantictumab + nab-paclitaxel + gemcitabine	Terminated
Ryan 2016 (NCT02013154) [40]	DKN-01	Monoclonal antibody against DKK1	Phase I; Non-randomized; Parallel	Recurrent or metastatic esophageal cancer, gastro-esophageal junction cancer	DKN-01; DKN-01 vs paclitaxel; DKN-01 vs pembrolizumab	Ongoing
Eads 2016 (NCT02375880) [41]	DKN-01	Monoclonal antibody against DKK1	Phase I; Single group	Advanced cholangiocarcinoma	DKN-01 + gemcitabine + cisplatin	Ongoing
NCT03645980	DKN-01	Monoclonal antibody against DKK1	Phase II; Non-randomized; Sequential	Advanced hepatocellular carcinoma	DKN-01 vs sequential DKN-01 + sorafenib	Recruiting
NCT04166721	DKN-01	Monoclonal antibody against DKK1	Phase II; Single group	Advanced gastroesophageal adenocarcinoma	DKN-01 + atezolizumab	Recruiting
Bendell 2016 (NCT02482441) [42]	Rosmantuzumab (OMP-131R10)	Monoclonal antibody against RSPO3	Phase I; Single group	Advanced solid malignancies, including metastatic colorectal cancer, etc.	OMP-131R10	Completed
NIKOLO trial (NCT02519582) [43]	Niclosamide	FZD1 inhibitor, LRP6 inhibitor	Phase II; Single group	Progressed colorectal cancer	Niclosamide	Recruiting
NCT02687009	Niclosamide	FZD1 inhibitor, LRP6 inhibitor	Phase I; Single group	Colorectal adenocarcinoma	Niclosamide	Terminated
NCT02726334	BNC101	Monoclonal antibody against LGR5	Phase I; Single group	Metastatic colorectal cancer	BNC101; BNC101+ FOLFIRI	Terminated
NCT01777477	Chloroquine	v-ATPase inhibitor	Phase I; Single group	Advanced pancreatic adenocarcinoma	Chloroquine + gemcitabine	Completed
Molenaar 2017 (NCT02496741) [44]	Chloroquine	v-ATPase inhibitor	Phase II; Single group	Advanced solid malignancies, including intrahepatic cholangiocarcinoma	Chloroquine + metformin	Completed
NCT01006369	Hydroxy-chloroquine	v-ATPase inhibitor	Phase II; Non-randomized; Parallel	Metastatic colorectal carcinoma	Hydroxychloroquine + FOLFOX6 + bevacizumab vs Hydroxychloroquine + XELOX + bevacizumab	Completed
Mahalingam 2014 (NCT01023737) [45]	Hydroxy-chloroquine	v-ATPase inhibitor	Phase I; Single group	Advanced solid malignancies, including colorectal cancer, etc.	Hydroxychloroquine + vorinostat	Completed
Boone 2015 (NCT01128296) [46]	Hydroxy-chloroquine	v-ATPase inhibitor	Phase II; Single group	Unresectable pancreatic ductal adenocarcinoma	Hydroxychloroquine + gemcitabine	Completed
Loaiza-Bonilla 2015 (NCT01206530) [47]	Hydroxy-chloroquine	v-ATPase inhibitor	Phase II; Single group	Advanced colorectal adenocarcinoma	Hydroxychloroquine + FOLFOX + bevacizumab	Completed
Wolpin 2014 (NCT01273805) [48]	Hydroxy-chloroquine	v-ATPase inhibitor	Phase I; Single group	Metastatic pancreatic cancer	Hydroxychloroquine	Completed
Hong 2017 (NCT01494155) [49]	Hydroxy-chloroquine	v-ATPase inhibitor	Phase II; Single group	Early pancreatic ductal carcinoma	Short course radiation therapy preoperatively. Hydroxychloroquine + capecitabine postoperatively	Ongoing
Karasic 2019 (NCT01506973) [50]	Hydroxy-chloroquine	v-ATPase inhibitor	Phase II; Randomized; Parallel	Advanced pancreatic adenocarcinoma	Hydroxychloroquine + nab-paclitacel + gemcitabine vs nab-paclitacel + gemcitabine	Ongoing
NCT01978184	Hydroxy-chloroquine	v-ATPase inhibitor	Phase II; Randomized; Parallel	Resectable pancreatic adenocarcinoma	Hydroxychloroquine + nab-paclitacel + gemcitabine vs nab-paclitacel + gemcitabine	Completed
NCT02013778	Hydroxy-chloroquine	v-ATPase inhibitor	Phase II; Single group	Unresectable hepatocellular carcinoma	Hydroxychloroquine + transarterial chemoembolization	Terminated
Arora 2019 (NCT02316340) [51]	Hydroxy-chloroquine	v-ATPase inhibitor	Phase II; Randomized; Crossover	Metastatic colorectal cancer	Hydroxychloroquine + vorinostat vs regorafenib	Completed
NCT03037437	Hydroxy-chloroquine	v-ATPase inhibitor	Phase II; Non-randomized; Parallel	Advanced hepatocellular cancer	Hydroxychloroquine + sorafenib vs sorafenib	Ongoing
NCT03215264	Hydroxy-chloroquine	v-ATPase inhibitor	Phase II; Single group	Metastatic colorectal cancer	Hydroxychloroquine + entinostat + regorafenib	Suspended
NCT03344172	Hydroxy-chloroquine	v-ATPase inhibitor	Phase II; Randomized; Parallel	Resectable pancreatic adenocarcinoma	Hydroxychloroquine + gemcitabine + nab-paclitaxel + avelumab vs hydroxychloroquine + gemcitabine + nab-paclitaxel	Suspended
NCT03377179	Hydroxy-chloroquine	v-ATPase inhibitor	Phase II; Single group	Advanced cholangiocarcinoma	ABC294640; Hydroxychloroquine + ABC294640	Ongoing
NCT03825289	Hydroxy-chloroquine	v-ATPase inhibitor	Phase I; Single group	Advanced pancreatic cancer	Hydroxychloroquine + trametinib	Ongoing
NCT04132505	Hydroxy-chloroquine	v-ATPase inhibitor	Phase I; Single group	KRAS-mutated metastatic pancreatic adenocarcinoma	Hydroxychloroquine + binimetinib	Ongoing
NCT04145297	Hydroxy-chloroquine	v-ATPase inhibitor	Phase I; Single group	MAPK-mutated GI cancers	Hydroxychloroquine + ulixertinib	Ongoing
NCT04214418	Hydroxy-chloroquine	v-ATPase inhibitor	Phase II; Non-randomized; Sequential	KRAS-mutated advanced solid malignancies, including pancreatic adenocarcinoma, colorectal adenocarcinoma, etc.	Hydroxychloroquine + atezolizumab + cobimetinib	Ongoing
El-Khoueiry 2013 (NCT01302405) [52]	PRI-724	β-catenin/CREBBP inhibitor	Phase I; Single group	Advanced solid malignancies, including colorectal cancer, etc.	PRI-724	Terminated
Ko 2016 (NCT01764477) [53]	PRI-724	β-catenin/CREBBP inhibitor	Phase I; Single group	Recurrent or advanced pancreatic adenocarcinoma	PRI-724 + gemcitabine	Completed
NCT02413853	PRI-724	β-catenin/CREBBP inhibitor	Phase II; Randomized; Parallel	Metastatic colorectal adenocarcinoma	mFOLFOX6/Bevacizumab + PRI-724 vs mFOLFOX6/Bevacizumab	Withdrawn
NCT03355066	SM08502	CLK inhibitor	Phase I; Single group	Advanced solid malignancies, including pancreatic cancer, colorectal cancer, etc.	SM08502	Recruiting

**Table 3:** All potential agents inhibiting Wnt signaling.

Mechanism	Agents
PORCN inhibitor	CGX1321, ETC-159, LGK974, GNF-6231, IWP-2, IWP-3, IWP-4, IWP-12, IWP-L6, IWP-O1, RXC004, WNT-C59
SFRP1 inhibitor	WAY-316606
*SFRP2* silencer inhibitor	Genistein
WNT5A mimic	Foxy-5
WNT inhibitor	Ant1.4Br/Ant1.4Cl, wogonin
WNT decoy receptor	Ipafricept
WNT3A-LRP5 complex inhibitor	APCDD1
FZD inhibitor	Vantictumab
FZD1&LRP6 inhibitor	Niclosamide
FZD4 inhibitor	FzM1
FZD7 inhibitor	Fz7-21
FZD10 inhibitor	OTSA101, OTSA101-DTPA-90Y
LGR5 inhibitor	BNC101
LRP6 inhibitor	Gigantol, salinomycin
FZD8-LRP6 heterodimer inhibitor	IGFBP-4
DKK1 inhibitor	DKN-01
DVL-PDZ domain inhibitor	Compound 3289-8625, FJ9, NSC668036, peptide Pen-N3
RSPO3 inhibitor	Rosmantuzumab
TNKS inhibitor	2X-121, AZ1366, AZ-6102, G007-LK, G244-LM, IWR-1, JW55, JW67, JW74, K-756, MN-64, MSC2504877, NVP-TNKS656, RK-287107, TC-E5001, WIKI4, XAV939
v-ATPase inhibitor	Apicularen, archazolid, bafilomycin, chloroquine, chondropsine, concanamycin, cruentaren, disulfiramthe, FR167356, FR177995, FR202126, hydroxychloroquine, indolyl, KM91104, lobatamide, NiK12192, oximidine, salicylihamide, SB 242784, tributyltin chloride
CK1 activator	Pyrvinium
GSK3β fragment mimic	TCS 183
β-catenin inhibitor	21H7, isoquercitrin, KY1220, KYA1797K, triptonide (NSC 165677, PG 492)
β-catenin degrader	MSAB, NRX-252114
β-catenin/TCF inhibitor	BC21, BC2059, CCT031374, CCT036477, CGP049090, CWP232228, ethacrynic acid, FH535, iCRT3, iCRT5, iCRT14, LF3, NLS-StAx-h, PKF115-584, PKF118-310, PKF118-744, PNU-74654, quercetin, ZTM000990
TNIK inhibitor	KY-05009, NCB-0846
β-catenin/EP300 inhibitor	IQ-1, windorphen, YH249/250
β-catenin/CREBBP&EP300 inhibitor	C-82, ICG-001, PRI-724, retinoids, vitamin D3
β-catenin/PYGO inhibitor	Pyrvinium
β-catenin/BCL9 inhibitor	Compound 22, carnosic acid, SAH-BCL9
CLK inhibitor	SM08502
Wnt/β-catenin signaling inhibitor	Adavivint (SM04690, lorecivivint), artesunate, cardamonin, cardionogen, CCT031374, diethyl benzylphosphonate, echinacoside, KY02111, pamidronic acid, specnuezhenide
